# How effective is nutrition training for staff running after school programs in improving quality of food purchased and meal practices? A program evaluation

**DOI:** 10.1186/s13104-024-06798-5

**Published:** 2024-05-14

**Authors:** Cecilie Beinert, Margrethe Røed, Frøydis N. Vik

**Affiliations:** Department of Nutrition and Public Health, University of Agder, Postboks 422, Kristiansand, 4604 Norway

**Keywords:** After school program, Food, Nutrition, Nutrition training, Intervention, Students, Norway

## Abstract

**Objectives / purpose:**

After school programs represents a setting for promoting healthy dietary habits. The aim of this study was to evaluate how effective the after school program staff perceived nutrition training aiming to improve quality of food purchased and meal practices. We further aimed to assess the changes in purchase of primarily fish and fish products, whole grains and fruit and vegetables, by collecting receipts from food purchase before and after the intervention.

**Results:**

This is a mixed methods study. Group interviews with after school staff were carried out and the data was analyzed deductively according to the RE-AIM framework. Receipts from food purchase were collected. Findings from the qualitative interviews indicated that the intervention had been a positive experience for the staff and suggested a new way of working with promoting healthy foods in after school program units. Although there were some challenges reported, the staff made necessary adjustments to make the changes possible to sustain over time. Findings from the receipts support the changes reported by the staff. These showed increased purchase of vegetables, fish, and whole grain in all four after school program units. After school programs in similar settings may expand on these findings to improve the students’ dietary habits.

**Supplementary Information:**

The online version contains supplementary material available at 10.1186/s13104-024-06798-5.

## Introduction

Investing in young children’s health, education, and development are fundamental for the individual’s lifelong health and development [1]. An unhealthy diet during childhood tends to track into adulthood [2] and increases the risk of childhood obesity [3] and non-communicable diseases later in life [4]. Developing healthy eating habits in childhood is therefore essential to maintain good health throughout life [5].

In Norway, the diet of children and adolescents (age 9 and 13) are mostly in line with national dietary guidelines, however, they still consume too much saturated fat and added sugar and too little fruit, vegetables and fish [6]. Parents play an important role in establishing healthy eating habits in their children, but arenas where children spend a considerable amount of their time and consume meals on a regular basis also influence eating habits. Schools and afterschool programs (ASPs), both represent areas of health promotion opportunities, as they amongst others, shall facilitate daily mealtimes that give the students a basis for developing enjoyment of food, a sense of community and good health habits [7]. Although the national guidance for ASPs is clear that they shall ensure the food served is in accordance with national dietary guidelines, previous investigations show this may not always be the case [8, 9], although the evidence base regarding foods served in ASPs in Norway is scarce. Every ASP has one leader and several members of staff. There are also no formal competence requirements for staff working in ASPs in Norway [10]. In 2021, 6 out of 10 students attended ASPs and it is most common for students in first grade (83%), with descending numbers up to fourth grade (30%) [11]. In 2022, the Norwegian government introduced up to 12 h per week free ASP for all first graders, resulting in an increase of participants from 83 to 92% [12]. As of August 1st, 2023, the government further introduced a 12 h per week free of charge ASP for second grade [13].

### The intervention

A non-profit foundation, Geitmyra Culinary Center for Children (from now on Geitmyra), has developed an intervention; a nutrition training, to enhance the competence of those working with food in ASPs, aiming to improve the quality of foods and meal practice within the setting and context of the ASP. Examples of context may be time available for preparing meals, amount of money allocated to food purchases, and if the kitchen facilities allow for preparing of different dishes).

The intervention was carried out in autumn 2022. The staff at the ASPs were to serve food in accordance with national dietary guidelines, implementing changes over time, and maintain the dietary changes over time. The schools were public and recruited through the municipality and they volunteered to serve as pilot schools to inform a scale up to include the whole municipality.

The intervention lasted for five months consisting of different components, e.g. initial meetings, menu planning and cooking courses and a “food celebration”. See Table 1 for details.

**Table 1:** Components of the Geitmyra culinary center for children nutrition training in after school programs (ASP)

Time	Nutrition training items lead by Geitmyra Culinary Center for Children	Participants
June 2022	Information meeting to motivate for participation (digital)	The municipalities leader of the school sector and advisers for ASP
	*Internal information*	The advisors inform the leaders of ASP and recruited four schools
August	Startup meeting	Principals and ASP-leader
	Startup meeting at the schools	ASP-leader and staff
September/ October	Mapping meeting at the schools	ASP-leader and staff
	Menu planning meeting at the schools	ASP-leader and staff
	Inspiration course at Geitmyra location	ASP staff and leaders
	Cooking course for the oldest students at Geitmyra location	Students, ASP staff and leaders
	+ 6 visits at the schools for counseling	ASP staff or sometimes ASP-leader
November	“Food celebration” at the school	students, ASP staff, and parents

Considering Norwegian student’s non-optimal diet and the need to increase ASP staff’s competence in preparing healthy meals served to students, our research questions were:

1. How effective did the ASP staff perceive the nutrition training to be?
2. How effective was the nutrition training targeting ASP staff in improving quality of foods purchased?

### Aim

The aim of this study was to evaluate how effective the ASP staff perceived nutrition training aiming to improve quality of food purchased and meal practices. Further, to assess the changes in purchase of primarily fish and fish products, whole grains and fruit and vegetables, by collecting receipts from ASPs food purchases before and after the intervention.

## Methods and materials

### Study design

To answer research question 1, the process evaluation, a qualitative study was conducted. Semi-structured group interviews were chosen as the data collection method because it gives comparable, reliable data and the flexibility to ask follow-up questions to clarify answers [14].

To answer research question 2, the outcome evaluation, a before and after comparison of quantitative data; foods purchased by ASPs were used. This was to test the data collection method, not powered to detect statistically significant change.

### Sample and procedure

Process evaluation: All ASP leaders from the participating ASPs were invited to take part in a digital group interview. There were four participants, all female and they agreed to participate by written consent. It lasted for 55 min. A semi-structured interview guide [15] exploring general experiences of the nutrition training was developed in addition to possible barriers and facilitators to implementation of the knowledge and skills. One ASP staff member (female) was interviewed individually, via zoom due to convenience and it lasted for 43 min. We were unable to recruit more participants among ASP staff. All interviews were conducted by two research staff, a research scientist (MR) and a master’s student (KDB), both of whom were women and teachers of food and health in a University in Southern Norway. Interviews were audio-recorded with consent from participants. Receipts of food purchases (objective measures) for all four ASP units were collected from January to May in 2022 and 2023, respectively.

### Data analysis – process evaluation

Interviews were transcribed verbatim (KDB). The transcripts were uploaded to NVivo 12 Pro. Data were analysed deductively to reflect participants perceptions of program impact as it related to the components of the RE-AIM framework. The RE-AIM framework was chosen to structure presentation of the process evaluation data because of its long history and broad utility [16, 17]. RE-AIM is a framework to guide the planning and evaluation of programs or interventions and involves assessment of program impact against five domains: Reach, Effectiveness, Adoption, Implementation, and Maintenance. Recordings were listened to several times by the lead researcher (CB) and data coded under the headings of the RE-AIM framework. Two other research members (FNV, MR) familiarized themselves with the material to check for similarities and differences between the researchers. Discrepancies were resolved through discussions.

### Data analysis - outcome evaluation

Grams per student per month were calculated from receipts for different food groups at both time points using Excel. Numbers from pure fish and fish products, whole grains, fruit, and vegetables are highlighted as these are of special interest because they were targeted in the intervention.

## Results

### Process evaluation – research question 1

How effective did the ASP staff perceive the nutrition training to be?

#### Reach

All ASP staff were invited to participate, and it was up to the individual ASP-leader to decide how many of their staff participated in the nutrition training. In total, 23 participated in the inspirational course (all four ASP leaders and 19 ASP staff in total from all four schools).

#### Effectiveness

A re-occurring feedback was that the intervention was a positive experience and that the intervention had provided them with valuable input. They reported changes in the way the meals are organized and that they included more vegetables. Instead of offering alternative dishes like bread with optional spread if students did not want the meal offered, they now offered cut vegetables or a single piece of bread as side dish to the meals prepared, to stimulate the students to try what’s offered; *“we have stopped always substituting with crispbread. But when serving soup, we either make crisp bread or they get a slice of whole grain bread. By that, we also learned that they don’t need so much, they get something. And you see that when they don’t get anything else, they try it instead”* (ASP leader 1).

A small group of the older students in the ASP were also trained during the intervention and helped select, prepare, and serve the meals, which was helpful to the employees who now experienced having more time for the students.

#### Adoption

The interviewees discussed how the intervention had been a positive experience. The recipes were possible to carry out with the facilities available. The dialogue with Geitmyra during the intervention had been positive and helpful. Still, a few challenges were reported. One school reported a challenge related to recruiting the older students to help prepare or serve the meal, due to few students in this age group and that most of them wanted to play or do other things. Challenges with left-over food were also reported by several, as the students did not like all the dishes that were served or that the portion sizes were too large. The staff worked hard to avoid food waste, by adjusting the portions sizes. They also replaced recipes with those the students liked, giving leftovers to the teachers in school, and storing the food and using it the day after, or later that day. As one ASP leader expressed *“We are fortunate to always have teachers who are hungry in the afternoon, so we usually get rid of the food …. The last time we had lentil soup, I heated it up and put it out when the parents came to pick them up. So, they got to taste it, but we, I also struggle making… its still a bit… too much, so we get a bit of leftovers”* (ASP leader 3).

#### Implementation

The interviewees expressed how they adjusted the intervention to make it sustain over time. This was partly related to barriers like time and personnel *“… we make it happen, but not to the extent that we did perhaps when Geitmyra was there with two extra adults…*” (ASP leader 2). One leader expressed how they used extra ovens in the school kitchen for preparing large quantities of crispbread for instance. Another ASP leader said they took the older students, who helped prepare the food, out of class a bit earlier, so they got enough time to prepare everything. Some also expressed how they skipped recipes their students did not like, instead of reintroducing it for repeated exposure. Some also adjusted the recipes to what the students preferred, by e.g., reducing the number of spices or by exchanging ingredients. There were no issues reported regarding economy related to purchase of food or equipment.

#### Maintenance

During the interviews, all agreed that they now had the tools needed to sustain these changes over time. As one ASP leader expressed, “*I really feel we got everything we need and more to continue*” (ASP leader 4). The ASP staff member expressed how she believed they would manage to continue, but that this required sustained initiative from their ASP leader. Post-intervention, the interviewees expressed how they had changed their practice.

### Outcome evaluation – research question 2

How effective was the nutrition training targeting ASP staff in improving quality of foods purchased?

Food purchases before and after the nutrition training were compared. In all four ASP-units there was an increase in the purchase of vegetables, pure fish, and whole grains (Table 2). The purchase of fruit decreased in two units and increased in the other two units. For results on other food groups relevant regarding dietary guidelines, see appendix 1.

**Table 2:** Food purchase in grams per student per month

	School 1		School 2		School 3		School 4	
	2022	2023	2022	2023	2022	2023	2022	2023
Vegetables	273	**544**	156	**457**	192	**555**	67	**367**
Fruit	158	**45**	274	**109**	26	**111**	51	**63**
Pure fish	0	**19**	12	**39**	0	**66**	0	**44**
Fish spreads/fish products	62	**17**	15	**6**	89	**8**	30	**47**
Legumes/pulses	0	**7**	0	**5**	0	**13**	0	**0**
Whole grain	254	**481**	446	**644**	677	**784**	183	**336**
White grain	106	**95**	119	**193**	60	**60**	66	**124**
Vegetables and fruit combined	431	**589**	430	**567**	218	**666**	118	**430**
Fish and fish products combined	62	**36**	27	**45**	89	**74**	30	**90**

## Discussion

This study aimed to investigate if nutrition training was effective in improving the skills and knowledge among ASPs staff and if this may lead to changes in food purchase in line with national dietary guidelines, which could improve the diet of students in ASPs. All four ASP units; 23 participants, did the nutrition training, which is considered decent, because ASP staff also needed to be with the students, and some were absent from work (reach).

Findings from the interviews indicated that the intervention had been a successful way of working with promoting healthy nutrition in ASPs (effectiveness). Although the staff met some challenges (adoption) and made some adjustments (implementation) to the intervention, they reported on changes made regarding student involvement and what they are serving. The ASP staff (including the leaders) reported being confident in continuing with the changes they had made, which may indicate a higher confidence and competence in their work regarding mealtimes (maintenance). Although there were challenges met regarding time and facilities, the staff made necessary adjustments to make it work.

The changes reported by the ASP staff and leaders were supported by the receipts collected pre- and post-interventions. For two ASPs, fruit purchase went down after the intervention. The ASP leader at school 1 said they had almost stopped serving fruit, and only served vegetables after the intervention, and school 4 mentioned how they had begun serving vegetables to all meals. This might explain the drop in fruit purchase at school 1 and the large rise in vegetable purchases at school 4. When assessing fruit and vegetables combined, this increased in all schools.

Mozaffarian et al. evaluated an organizational intervention in after school programs in the US, to improve snack and beverage quality [18]. They found significant improvements with respect to increasing servings of fresh fruits and vegetables [18] in line with our study regarding vegetables, and partly for fruits. They did not find increased servings of whole grains as we did in our study. Since this study evaluated private ASPs in the US, we cannot assume that settings and context are similar to our Norwegian study. To our knowledge, there are sparse literature available in a Nordic context for interventions targeting nutrition among ASP staff. Our findings from this study may support implementation of a more comprehensive training program in Norway and may also inform after school programs in a Nordic setting.

This study found that nutrition training with close follow-up over time may be an effective way of creating change in food and meal practices in ASPs. There are large societal benefits to be made if youth in Norway adhere to dietary guidelines, such as prevention of non-communicable diseases and economic benefits [19] and investing in child health will, therefore, yield long term benefits [1, 2, 4]. Although there are dietary guidelines on food offered in ASPs in Norway, these must also be followed by the individual units, which may not always be the case [8, 9].

## Conclusion

Findings from this program evaluation of nutrition training showed that it was effective in improving skills and knowledge among after school program staff. Receipts from food purchases before and after the intervention revealed an increase in purchase of vegetables, pure fish, and whole grain products.

### Limitations

Although all four ASP leaders agreed to participate in group interviews, the number of ASP units was small, and we were only able to interview one ASP staff. More participants would have made it possible to assess effectiveness to a larger degree. No control group was included, and we cannot rule out that changes in food purchases could be explained by other factors besides the intervention. Also, there is no long-term follow-up of the participants. Strengths include purchasing data (receipts) collected over a period of five months before and after the staff training, which are objective data showing change. The fact that the receipts were collected the same five months (January to May) the two consecutive years, limits season variation in food purchase and strengthen comparison. Strengths are also in applying mixed methods.

### Electronic supplementary material

Below is the link to the electronic supplementary material.

**Supplementary Material 1**: Appendix 1 Food purchase in grams per student per month

## Data Availability

The datasets used and/or analyzed during the current study are available from the corresponding author on reasonable request.

## Abbreviations

ASP: After school program
Geitmyra: Geitmyra Culinary Center for Children
