# Supplementary material for: How effective is nutrition training for staff running after school programs in improving quality of food purchased and meal practices? A program evaluation
Source: BMC Res Notes. 2024 May 14;17:136. doi: 10.1186/s13104-024-06798-5 (PMC11094912; doi:10.1186/s13104-024-06798-5)
Supplement: Supplementary file 1 — Supplementary Material 1: Appendix 1 Food purchase in grams per student per month [file 13104_2024_6798_MOESM1_ESM.docx]

|  | School 1 | | School 2 | | School 3 | | School 4 | |
| --- | --- | --- | --- | --- | --- | --- | --- | --- |
|  | 2022 | **2023** | 2022 | **2023** | 2022 | **2023** | 2022 | **2023** |
| Vegetables | 273 | **544** | 156 | **457** | 192 | **555** | 67 | **367** |
| Fruit | 158 | **45** | 274 | **109** | 26 | **111** | 51 | **63** |
| PURE Fish | 0 | **19** | 12 | **39** | 0 | **66** | 0 | **44** |
| Fish spreads*/FISH PRODUCTS | 62 | **17** | 15 | **6** | 89 | **8** | 30 | **47** |
| LegumEs/pulses | 0 | **7** | 0 | **5** | 0 | **13** | 0 | **0** |
| WHole grain | 254 | **481** | 446 | **644** | 677 | **784** | 183 | **336** |
| white Grain | 106 | **95** | 119 | **193** | 60 | **60** | 66 | **124** |
| Meat | 90 | **49** | 115 | **85** | 104 | **48** | 41 | **76** |
| Meat spreads/processed | 28 | **41** | 22 | **35** | 249 | **226** | 24 | **40** |
| Other spread | 12 | **7** | 21 | **26** | 0 | **0** | 0 | **0** |
| Cheese | 66 | **109** | 192 | **240** | 146 | **141** | 45 | **61** |
| Egg | 23 | **20** | 5 | **4** | 4 | **11** | 8 | **10** |
| Whole- and semi processed products | 10 | **15** | 53 | **49** | 0 | **0** | 16 | **26** |
| Butter and oils | 17 | **28** | 25 | **88** | 49 | **43** | 28 | **31** |
| Sweets and snacks | 164 | **19** | 79 | **73** | 49 | **49** | 10 | **21** |
| Ketchup | 38 | **15** | 47 | **25** | 98 | **37** | 13 | **25** |
| Juice | 22 | **71** | 3 | **35** | 12 | **35** | 0 | **15** |
| Potato** | 12 | **5** | 0 | **5** | 11 | **25** | 23 | **16** |
| Soft drinks and lemonade | 0 | **0** | 28 | **12** | 69 | **18** | 0 | **0** |
|  |  |  |  |  |  |  |  |  |
| Vegetables and fruit | 431 | **589** | 430 | **567** | 218 | **666** | 118 | **430** |
| Fish and fish products | 62 | **36** | 27 | **45** | 89 | **74** | 30 | **90** |
| Meat and meat products | 118 | ***90*** | 137 | ***120*** | 353 | ***275*** | 65 | ***117*** |

Appendix 1 Food purchase in grams per student per month.

* Fish spread: fish based spread for sandwiches, e.g. mackerel in tomato sauce

**In Norway, potato is not defined as a vegetable, and is therefore mentioned separately.
